# Klinische Erfahrungen über Diabetes Mellitus

**Published:** 1900-03

**Authors:** 


**Klinische Erfahrungen über Diabetes Mellitus.** By von Dr. E. KÜLZ, Prof. Drs. TH. RUMPF, G. ALDEHOFF und W. SANDMEYER. Pp. xiv., 552. Jena: Gustav Fischer. 1899.

When Dr. Külz died in 1895, he left behind him an enormous collection of observations and records of cases of diabetes. Rumpf, who had worked with him, was requested to utilise them. He has published in this volume the history of 692 patients, with a most elaborate analysis of the results of treatment and discussions of the chief complications and symptoms of the disease. The volume forms an important contribution to our knowledge, and one which will have a permanent influence. Such a mass of facts has probably never before been collected, and no subsequent writers can afford to overlook them. Külz took the view that there is only one kind of diabetes, and that all the apparently different forms can be resolved into variations of severity and individual resistance under identical disturbances. The chief point in the treatment he adopted was dietetic management, adapted to each individual so as to increase the power of assimilating starch. Thus the effects of each change from the strictest to ordinary diet on the excretion of sugar is recorded, together with the presence of acetone, and the quantity of ammonia, urea, and albumin. The amount of labour which has been employed in this monumental work must have been enormous. The essays on kidney and other complications of diabetes are even by themselves elaborate monographs.

